# Genome-wide analyses of long noncoding RNA expression profiles correlated with radioresistance in nasopharyngeal carcinoma via next-generation deep sequencing

**DOI:** 10.1186/s12885-016-2755-6

**Published:** 2016-09-06

**Authors:** Guo Li, Yong Liu, Chao Liu, Zhongwu Su, Shuling Ren, Yunyun Wang, Tengbo Deng, Donghai Huang, Yongquan Tian, Yuanzheng Qiu

**Affiliations:** 1 Department of Otolaryngology Head and Neck Surgery, Xiangya Hospital, Central South University, Xiangya Road, Changsha, 410008 Hunan China; 2 Otolaryngology Major Disease Research Key Laboratory of Hunan Province, Xiangya Road, Changsha, 410008 Hunan China

**Keywords:** Nasopharyngeal carcinoma, Radioresistance, Long noncoding RNA, Deep sequencing

## Abstract

**Background:**

Radioresistance is one of the major factors limiting the therapeutic efficacy and prognosis of patients with nasopharyngeal carcinoma (NPC). Accumulating evidence has suggested that aberrant expression of long noncoding RNAs (lncRNAs) contributes to cancer progression. Therefore, here we identified lncRNAs associated with radioresistance in NPC.

**Methods:**

The differential expression profiles of lncRNAs associated with NPC radioresistance were constructed by next-generation deep sequencing by comparing radioresistant NPC cells with their parental cells. LncRNA-related mRNAs were predicted and analyzed using bioinformatics algorithms compared with the mRNA profiles related to radioresistance obtained in our previous study. Several lncRNAs and associated mRNAs were validated in established NPC radioresistant cell models and NPC tissues.

**Results:**

By comparison between radioresistant CNE-2-Rs and parental CNE-2 cells by next-generation deep sequencing, a total of 781 known lncRNAs and 2054 novel lncRNAs were annotated. The top five upregulated and downregulated known/novel lncRNAs were detected using quantitative real-time reverse transcription-polymerase chain reaction, and 7/10 known lncRNAs and 3/10 novel lncRNAs were demonstrated to have significant differential expression trends that were the same as those predicted by deep sequencing. From the prediction process, 13 pairs of lncRNAs and their associated genes were acquired, and the prediction trends of three pairs were validated in both radioresistant CNE-2-Rs and 6-10B-Rs cell lines, including lncRNA n373932 and *SLITRK5*, n409627 and *PRSS12*, and n386034 and *RIMKLB*. LncRNA n373932 and its related *SLITRK5* showed dramatic expression changes in post-irradiation radioresistant cells and a negative expression correlation in NPC tissues (*R* = −0.595, *p* < 0.05).

**Conclusions:**

Our study provides an overview of the expression profiles of radioresistant lncRNAs and potentially related mRNAs, which will facilitate future investigations into the function of lncRNAs in NPC radioresistance.

**Electronic supplementary material:**

The online version of this article (doi:10.1186/s12885-016-2755-6) contains supplementary material, which is available to authorized users.

## Background

Radiotherapy is the mainstay treatment for patients with nasopharyngeal carcinoma (NPC). Despite major advances in radiation techniques and radiotherapeutic strategies, the prognosis and survival rates of patients with NPC remain unsatisfactory [1, 2]. One of the major factors contributing to the poor outcomes in NPC is the occurrence of radioresistance, and therefore radioresistance is a hot topic in both basic and clinical research. Multiple studies have shown that radioresistance-associated molecules (mRNAs, microRNAs, and proteins) influence radioresistance by regulating radioresistance-associated processes, including DNA repair capacity, apoptosis, cell cycle arrest, and protective autophagy [3–5]. Previous studies have improved our understanding of radioresistance. However, the exact molecular mechanisms underlying radioresistance remain unclear.

Approximately 1.5 % of the entire human genome is involved in protein transcription and translation [6]. The majority of the remaining noncoding regulatory elements are transcribed into noncoding RNAs that have been implicated in a variety of human diseases including cancers. Long noncoding RNAs (lncRNAs) are a novel class of mRNA-like transcripts that have been shown to be involved in the development and progression of different cancers [7]. Moreover, studies analyzing large clinical cancer samples have demonstrated that certain lncRNAs (i.e., HOTAIR and MALAT1) serve as valuable prognostic biomarkers [8, 9]. Therefore, increasing studies have focused on the functions and mechanisms of lncRNAs in cancer malignant behaviors such as unlimited proliferation, migration, and metastasis [10, 11]. However, few investigations have focused on the association of lncRNAs with cancer radioresistance.

In an effort to improve our understanding of the mechanisms of radioresistance, we previously established NPC radioresistant cell lines by gradually increasing the dose of irradiation [11, 12]. Based on the potential roles proposed for lncRNAs in cancer-related behaviors, here we investigated the possible lncRNAs mediating NPC radioresistance. We compared NPC radioresistant cells and parental cells to obtain global lncRNA expression profiles associated with radioresistance using next-generation, high-throughput deep sequencing technology.

## Methods

### Cell lines and cell culture

The CNE-2 and 6-10B poorly differentiated NPC cell lines were purchased from the Cell Center of Central South University (Changsha, China). CNE-2-Rs and 6-10B-Rs cells, which exhibit a radioresistant phenotype, were established through exposure to gradually increasing levels of irradiation as previously described [12, 13]. All cells were propagated in RPMI 1640 medium (Hyclone, Logan, UT, USA) with 10 % fetal bovine serum (Gibco BRL, Gaithersburg, MD, USA) and 1 % antibiotics (Gibco BRL) and incubated at 37 °C with saturated humidity and 5 % CO_2_.

### Patient tissues

Forty-three NPC tissues were obtained from patients undergoing biopsy of the nasopharynx at the Department of Otolaryngology Head and Neck Surgery, Xiangya Hospital, Central South University from March 2014 to March 2015. All NPC patients had no history of radiotherapy or chemotherapy. All tissues were immediately snap frozen and stored in liquid nitrogen before total RNA extraction. Informed consent was obtained from all patients prior to the biopsy. The study was approved by the Research Ethics Committee of Central South University, Changsha, China.

### RNA extraction

Total RNA was extracted from NPC cells using TRIzol (Invitrogen, Carlsbad, CA, USA) according to the manufacturer’s protocol. Total RNA from the NPC radioresistant cell line CNE-2-Rs and its parental cell line CNE-2 was analyzed using the Agilent RNA 6000 Nano LabChip® kit with the Agilent 2100 Bioanalyser (Agilent Technologies, Palo Alto, CA, USA) to determine its quantity and integrity. According to criteria in the literature, only samples that scored more than 4 were considered to be reliable samples for the next sequencing step [14].

The RNA of other cells (i.e., 6-10B, 6-10B-Rs, NPC cells with or without irradiation) and NPC tissues were also extracted for the sequencing result validation. The concentration and integrity of the RNAs were determined using a Nano Drop 2000 Spectrophotometer (Thermo Scientific, Wilmington, DE, USA). RNA quality was determined by quantification of 28S and 18S ribosomal RNA on ethidium bromide (EB)-stained gels.

### Construction of a RNA library for sequencing

RNA was extracted from CNE-2 and CNE-2-Rs cells, and the Ribo-Zero™ Kit (Epicentre, Madison, WI, USA) was used to remove the rRNA. The remaining RNA was cut randomly into short fragments. First-strand cDNA was transcribed based on these random fragments using random hexamers; second-strand cDNA was transcribed by mixing the first-strand cDNA with buffer, dNTPs, RNase H, and DNA polymerase I. Short fragments were purified using the QIAquick PCR Purification Kit (Qiagen, Valencia, CA, USA) and resolved with EB buffer for end reparation and single nucleotide A (adenine) addition. The short fragments were connected with adapters and the second strand was degraded using UNG (uracil-N-glycosylase). RNA fragments were separated by agarose gel electrophoresis, and the fragments were expanded with polymerase chain reaction (PCR). The PCR products were sequenced using an Illumina HiSeq™ 2000 instrument (Illumina Inc., San Diego, CA, USA), and the original image data were converted into “.fq” files by base calling software. The relative data were submitted to NCBI under BioProject accession No. PRJNA 254709. The details of the experiment were as follows: expected library size: 200 bp; read length: 90 nt; and sequencing strategy: paired-end sequencing.

### Raw data filtering and rRNA removal

The raw reads were saved in the fastq format. We removed the dirty raw reads prior to data analysis. Three criteria were used to filter out dirty raw reads: 1) reads with adapters were removed; 2) reads in which unknown bases occurred with a frequency greater than 10 % were removed; and 3) low-quality reads (the percentage of low-quality bases was over 50 % in a read). Filtered reads were removed. The remaining reads were called “clean reads” and used for the bioinformatics analyses.

In consideration of rRNA pollution interference in the analysis, the clean reads were mapped to an rRNA reference sequence using the short reads alignment software *SOAP*2 (http://soap.genomics.org.cn/) to remove the remaining rRNA reads. The remaining reads were used for transcriptome assembly and quantification.

### Transcript reconstruction

The removed rRNA reads were mapped to a reference genome using an improved version of TopHat2 (http://ccb.jhu.edu/software/tophat/index.shtml) that could align reads across splice junctions without relying on gene annotation. We permitted two base pair mismatches in this step. Reads mapped to the genome were assembled by Cufflinks [15]. We used the Reference Annotation Based Transcripts algorithm to assemble the reads into transcripts.

### Known and unknown lncRNA identification

The transcripts were BLASTed against the NONCODE v3.0 database (http://www.noncode.org/NONCODERv3/) to identify known noncoding RNAs using the following selection criteria: identity > 0.9, coverage > 0.8, and E-value < 10^5^. These transcripts were named as the ID number in the NONCODE v3.0 database.

Transcripts without annotations in the ncRNA library were compared with protein databases such as the KEGG (Kyoto Encyclopedia of Genes and Genomes), nr (non-redundant amino acid database), COG (Cluster of orthologous Groups of proteins), and Swissprot Database, and the mapped transcripts were considered to be mRNA (identity > 0.9 and coverage > 0.8). The remaining transcripts that were not aligned with the protein library were inputted into the Coding Potential Calculator program to distinguish coding and noncoding sequences. A true protein-coding transcript is more likely to have a long and high-quality open reading frame (ORF) compared with a non-coding transcript. Here, we considered the following six features: log-odds score, coverage of the predicted ORF, integrity of the predicted ORF, number of HITs, hit score, and frame score (http://cpc.cbi.pku.edu.cn/).

### Establishment of differential expression profiles

To obtain the differential expression profiles, read counts and reads per kilobase per million reads (RPKM) values were calculated for each lncRNA. The formula was defined as below:

$$ \mathrm{RPKM}=\frac{1{0}^6\;\mathrm{C}}{\mathrm{NL}/10} $$

in which C was the number of reads that uniquely mapped to the given lncRNAs, N was the number of reads that uniquely mapped to all lncRNAs, and L was the total length of the lncRNA. For lncRNAs with more than one alternative transcript, the longest transcript was selected to calculate the RPKM. The RPKM method eliminates the influence of different lncRNA lengths and sequencing discrepancies on the lncRNA expression calculation. Therefore, the RPKM value can be directly used to compare differences in lncRNA expression among samples. Then, we identified differentially expressed lncRNAs between the radioresisitant NPC cell CNE-2-Rs and the radiosensitive parental cell CNE-2 based on the following criteria: false discovery rate ≤ 0.001 and fold change ≥ 2. NPC radioresistant mRNA differential expression profiles were reported in our previous study [11]. LncRNAs and mRNAs with differential expression were selected for the subsequent analyses.

### Prediction of mRNA-related lncRNAs

At present, there are no widely accepted standardized algorithms for the prediction of mRNAs potentially associated with lncRNAs. In current publications, the prediction strategies are primarily based on the mutual interaction between lncRNAs and their associated mRNAs; antisense and up/downstream prediction methods are two common strategies for the prediction of lncRNA-related mRNAs.

To examine antisense lncRNA-mRNA interactions, we searched all antisense lncRNA-mRNA duplexes with complementary base pairing using the RNAplex software with the ViennaRNA package [16, 17]. The antisense lncRNA and its target mRNA were selected according to the minimum free energy, complementary base coverage (>95 %), and opposing expression trends. Up/downstream lncRNA candidates were predicted by a *BLAST* search of lncRNA sequences in the flanking regions of the coding genes. We defined the flanking region as the sequence within 2 kb up- or downstream of the coding genes in which most of the regulatory elements were located.

### Real-time quantitative reverse transcription-PCR (qRT-PCR) validation of differentially expressed lncRNAs

Briefly, cDNA was transcribed from total RNA using the PrimeScript RT reagent kit with a DNA Eraser (TaKaRa, Shiga, Japan). Primers for lncRNAs were designed and synthesized. Then, qPCR assays were performed using a Bio-Rad IQTM5 Multicolor Real-Time qRT-PCR detection system (Bio-Rad, Hercules, CA, USA). The expression levels of lncRNAs and genes were detected using primers specific for the lncRNAs and mRNAs (Additional file 1: Table S1). Human *beta-actin* was used as a housekeeping gene for normalization. The expression levels of lncRNAs and mRNAs were measured in terms of the cycle threshold (CT) and normalized to *beta-actin* expression using the 2^-ΔΔCt^ method.

### Irradiation

Irradiation was delivered at room temperature with a 6-MeV electron beam generated by the linear accelerator 2100EX (Varian Medical, Inc., Palo Alto, CA, USA) at a dose rate of 300 cGy/min. A compensation glue with 1.5-cm thickness covered the cell culture containers. The source-to-skin distance was 100 cm.

### Statistical analysis

The results of the quantitative data in this study were expressed as the mean ± standard deviation. The statistical significance of the differences between two groups was analyzed using a two-sided unpaired Student’s *t* test (for equal variance) or Welch’s corrected *t* test (unequal variance). The correlation between lncRNA n373932 and SLITRK5 mRNA expressions was first made as a napierian logarithmic transformation (base number is approximately 2.7183) and then calculated using bivariate correlation analyses (Pearson correlation). The above analyses were performed with SPSS 18.0 software (IBM Corporation, Armonk, NY, USA). Differences with *P* values less than 0.05 were considered statistically significant.

## Results

### Construction of lncRNA profiles that correlated with NPC radioresistance

**Fig. 1:**
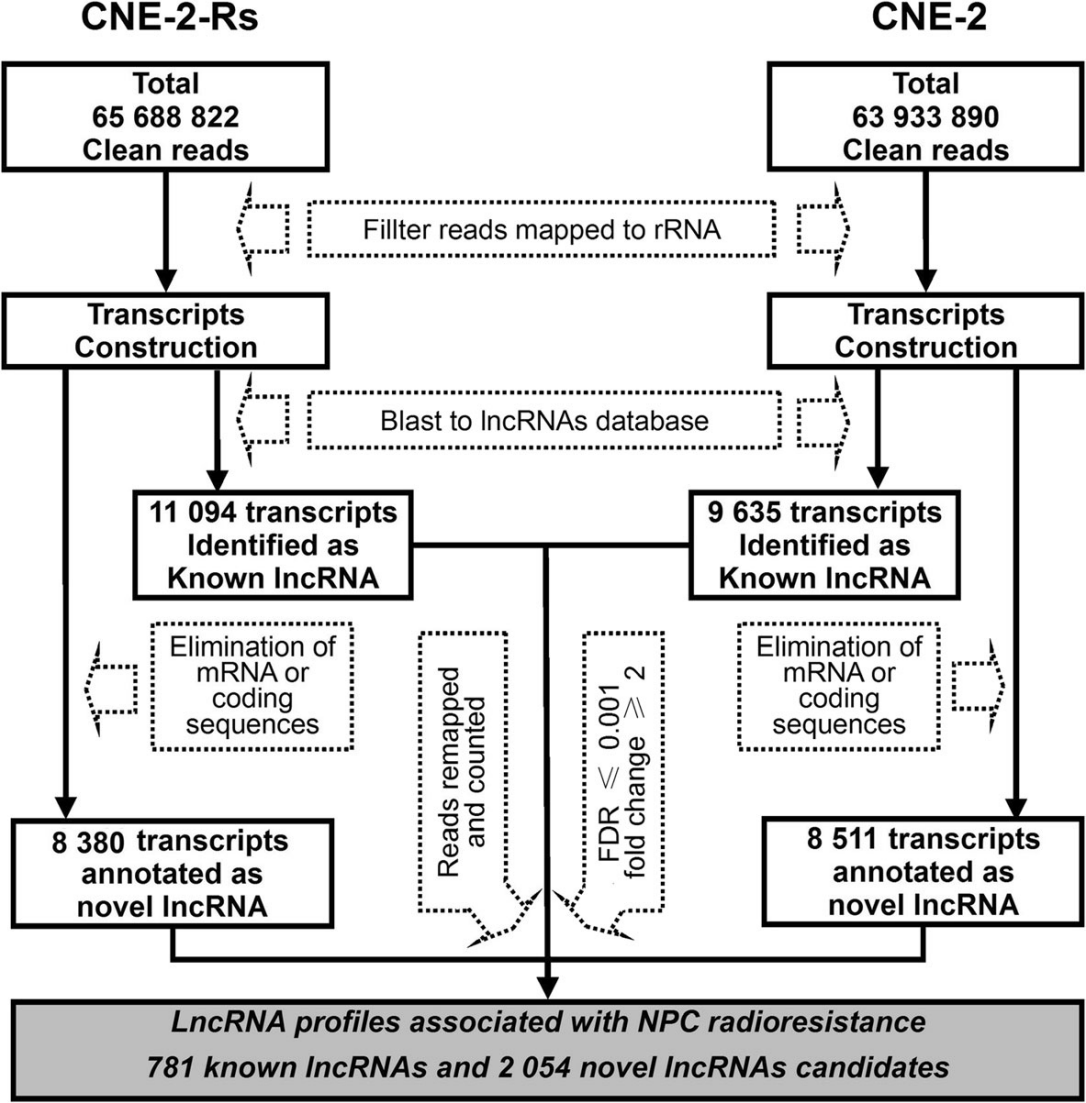
The work flow of constructing long noncoding RNA profiles correlated with NPC radioresistance

To obtain lncRNA expression profiles associated with radioresistance, we constructed cDNA libraries using our previously established radioresistant CNE-2-Rs and parental CNE-2 cell lines [11]. As depicted in Fig. 1, a total of 65,688,822 and 63,933,890 clean reads were obtained from the CNE-2-Rs and CNE-2 cells, respectively. After eliminating reads mapped to rRNA, TopHat2 and Cufflinks were used to reconstruct transcripts in both samples. The reconstructed transcripts were BLASTed against the NONCODE v3.0 database. A total of 11,094 transcripts in the CNE-2-Rs cells and 9,635 transcripts in the CNE-2 cells were annotated as known lncRNAs. Following elimination of transcripts mapped to mRNA and coding sequences, 8,380 (CNE-2-Rs) and 8,511 (CNE-2) transcripts were separately identified as novel lncRNAs. The unique mapped reads for each lncRNA were counted, and the RPKM values for each lncRNA were calculated. Based on the criteria of an absolute fold change > 2.0 and a false discovery rate < 0.001, 781 known lncRNAs and 2,054 novel lncRNA candidates were finally obtained. These lncRNAs constituted the differential lncRNA expression profiles associated with NPC radioresistance (Additional file 2: Table S2).

### Overview of the lncRNAs associated with NPC radioresistance

**Fig. 2:**
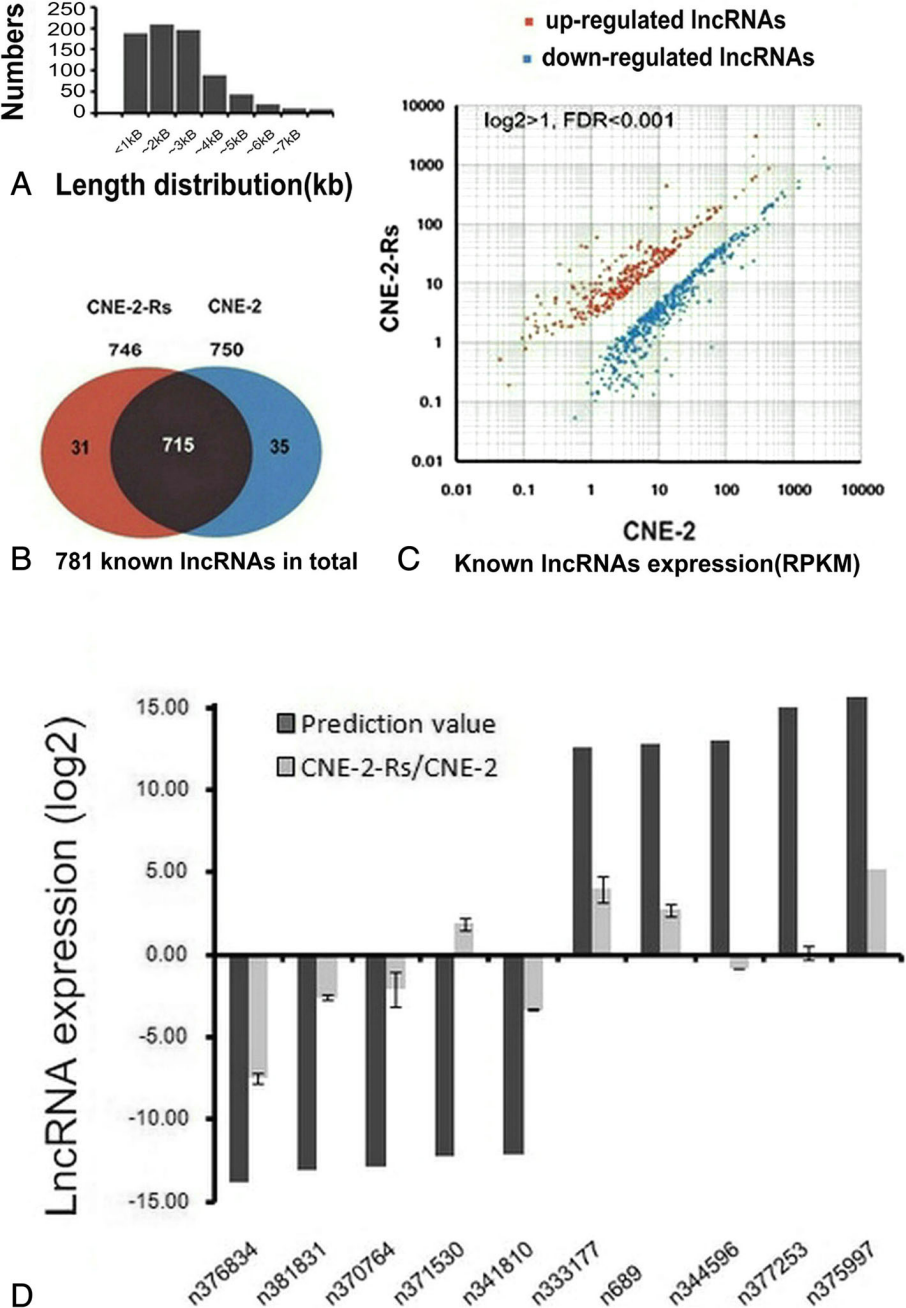
Overview of known lncRNAs associated with NPC radioresistance. **a** The length distributions of the transcripts. **b** The known lncRNAs identified in CNE-2 and CNE-2-Rs cells. The shaded area represented the lncRNAs found in both samples, and the areas coloured red and blue showed the number of lncRNAs expressed in samples CNE-2 and CNE-2-Rs, respectively. **c** Scatter plot of lncRNA expression profiles in radiosensitive CNE-2 (x-axis) and radioresistant CNE-2-Rs cells (y-axis). The significantly up-regulated lncRNAs were marked in red and the down-regulated lncRNAs in blue. |log2 Fold change| ≥ 1 and FDR < 0.001. **d** Validation of the expression levels of the top five up-regulated and down-regulated known lncRNAs via qRT-PCR

**Fig. 3:**
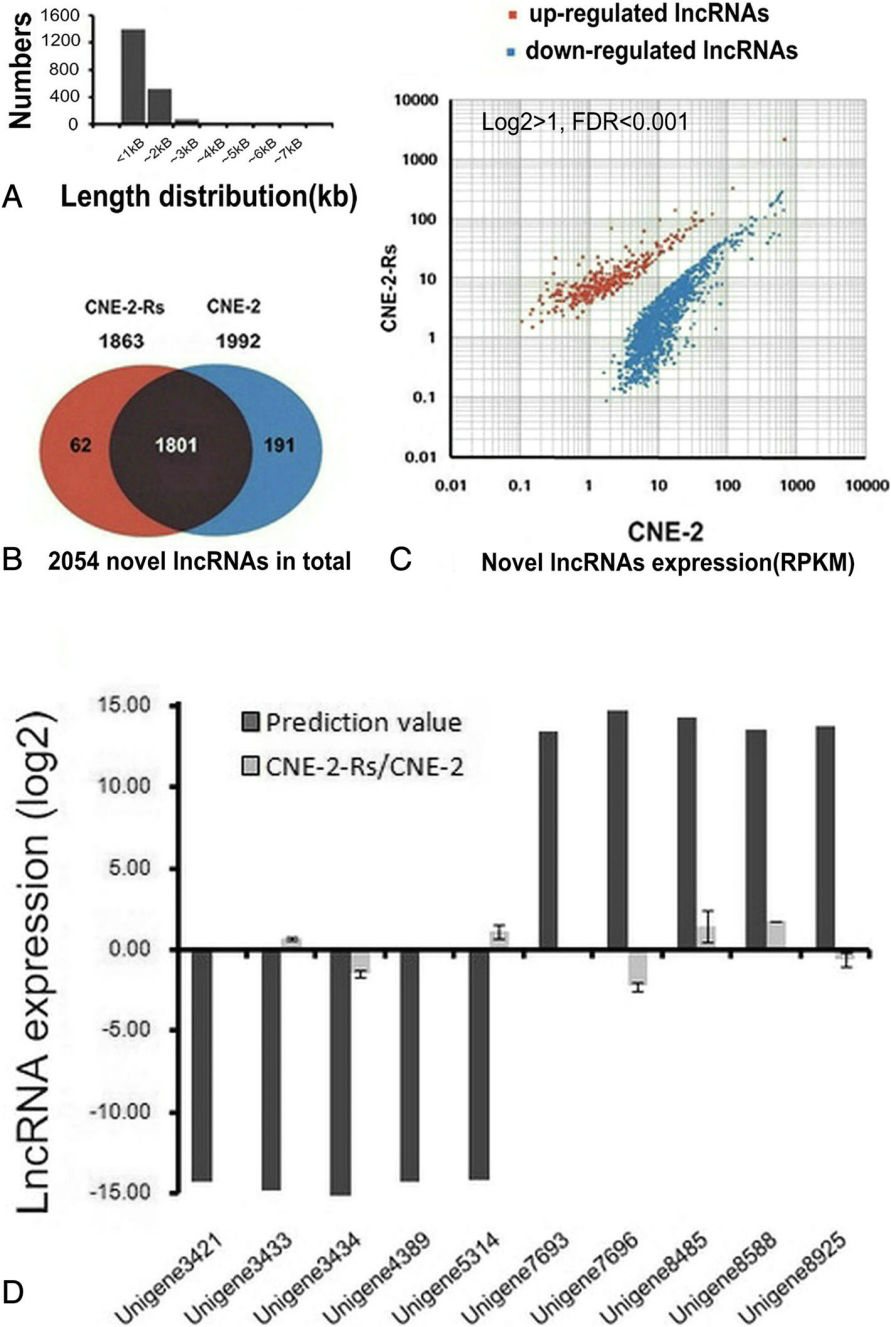
Overview of novel lncRNA candidates associated with NPC radioresistance. **a** The length distributions of the transcripts. **b** The novel transcripts identified in CNE-2 and CNE-2-Rs cells. The shaded areas represented the lncRNAs found in both samples, and the areas coloured red and blue showed the number of lncRNAs expressed in samples CNE-2 and CNE-2-Rs, respectively. **c** Scatter plot of lncRNA expression profiles in radiosensitive CNE-2 (x-axis) and radioresistant CNE-2-Rs cells (y-axis). The significantly up-regulated novel lncRNAs were marked in red and the down-regulated lncRNAs in blue. |log2 Fold change| ≥ 1 and FDR < 0.001. **d** Validation of the expression of the top five up-regulated and down-regulated novel transcripts via qRT-PCR

The features of the lncRNAs were analyzed based on the above-mentioned lncRNA profiles. Our data revealed that most known lncRNAs were 200 bp to 3 kb in length (Fig. 2a), while the novel candidates were mainly distributed between 200 bp and 2 kb (Fig. 3a). Among the 781 known lncRNAs, 715 lncRNAs were expressed in both cell lines and 31 and 35 were present only in CNE-2-Rs or CNE-2 cells, respectively (Fig. 2b). A total of 310 lncRNAs were upregulated and 471 lncRNAs were downregulated in the radioresistant CNE-2-Rs cells compared with the parental CNE-2 cells (Fig. 2c). Similarly, 1800 lncRNAs of the 2054 lncRNA candidates were expressed in both cells, while 192 and 62 were expressed only in CNE-2-Rs or CNE-2, respectively (Fig. 3b). A total of 406 lncRNAs were elevated and 1648 were decreased in the radioresistant CNE-2-Rs cells (Fig. 3c).

To confirm the consistency of the known and novel lncRNA expression profiles, the top five upregulated and downregulated lncRNAs in both the known and novel lncRNAs were identified. Our qRT-PCR assays verified that 7 of 10 known lncRNAs exhibited a significant differential expression with the same trend observed in the deep sequencing prediction, including the upregulated lncRNAs n333177, n689, and n375997 and the downregulated lncRNAs n376834, n381831, n370764, and n341810 (Fig. 2d). A total of 3 of 10 novel candidates displayed the same expression changes as the prediction, including Unigene3434, Unigene8485, and Unigene8588 (Fig. 3d). Our results indicated that the known lncRNAs had a higher matching ratio than the novel lncRNAs. We mainly focused on these known candidates in this study.

### Prediction of lncRNA and mRNA associations based on the antisense and up/downstream strategies

At present, there are no widely accepted standardized algorithms for the prediction of the potential association of mRNAs with lncRNAs. In current publications, the prediction strategies are primarily based on the mutual interaction method between lncRNAs and their associated mRNAs; the antisense and up/downstream prediction methods are two common strategies for the prediction of lncRNA and mRNA associations [18, 19]. Therefore, based on the radioresistant lncRNA profiles and mRNA profiles established in our previous work, we initially used the antisense prediction method to search all antisense lncRNA-mRNA duplexes via the RNAplex software. This program searches for complementary pairing between the bases of the lncRNAs and potential mRNAs. Two lncRNAs (n342800 and n341092) were found to be complementarily paired with the mRNAs *NFE2 L3* and *CHORDC1* (Table 1). The up/downstream prediction strategy was also used to BLAST the lncRNA sequences within the 2 kb upstream or downstream regions of the mRNA coding sequences. As shown in Table 1, 7 lncRNAs (n373932, n411012, n376260, n339364, n369600, n345672, and n367357) were found to bind to the upstream region of their associated mRNAs (*SLITRK5, MNX1, RAB3A, TWF2, PDK4, H1FX*, and *CCNG2*), and four lncRNAs (n409627, n386034, n410131, and n386687) were predicted to interact with the downstream domain of their potential mRNAs (*PRSS12, RIMKLB, ZNF783,* and *NEU3*). Taken together, the base-pairing prediction strategy provided 13 pairs of lncRNAs and associated mRNAs that served as clues to investigate their actual interactions and affections on NPC radioresistance.

**Table 1:** Antisense lncRNA and up/downstream-associated lncRNA prediction

lncRNA	Fold change	Gene name	Fold change	Origin
n342800	−2.21	NFE2L3	2.49	Anti-sense
n341092	2.29	CHORDC1	−1.31	Anti-sense
n409627	−2.07	PRSS12	9.93	Down-stream
n386034	−1.92	RIMKLB	−1.26	Down-stream
n410131	−1.71	ZNF783	−1.28	Down-stream
n386687	1.04	NEU3	1.08	Down-stream
n373932	−10.59	SLITRK5	1.93	Up-stream
n411012	1.37	MNX1	2.55	Up-stream
n376260	1.74	RAB3A	3.09	Up-stream
n339364	2.19	TWF2	1.16	Up-stream
n369600	2.52	PDK4	−1.89	Up-stream
n345672	3.52	H1FX	1.55	Up-stream
n367357	9.64	CCNG2	2.07	Up-stream

### Validation of the lncRNAs and their related mRNAs by qRT-PCR

**Fig. 4:**
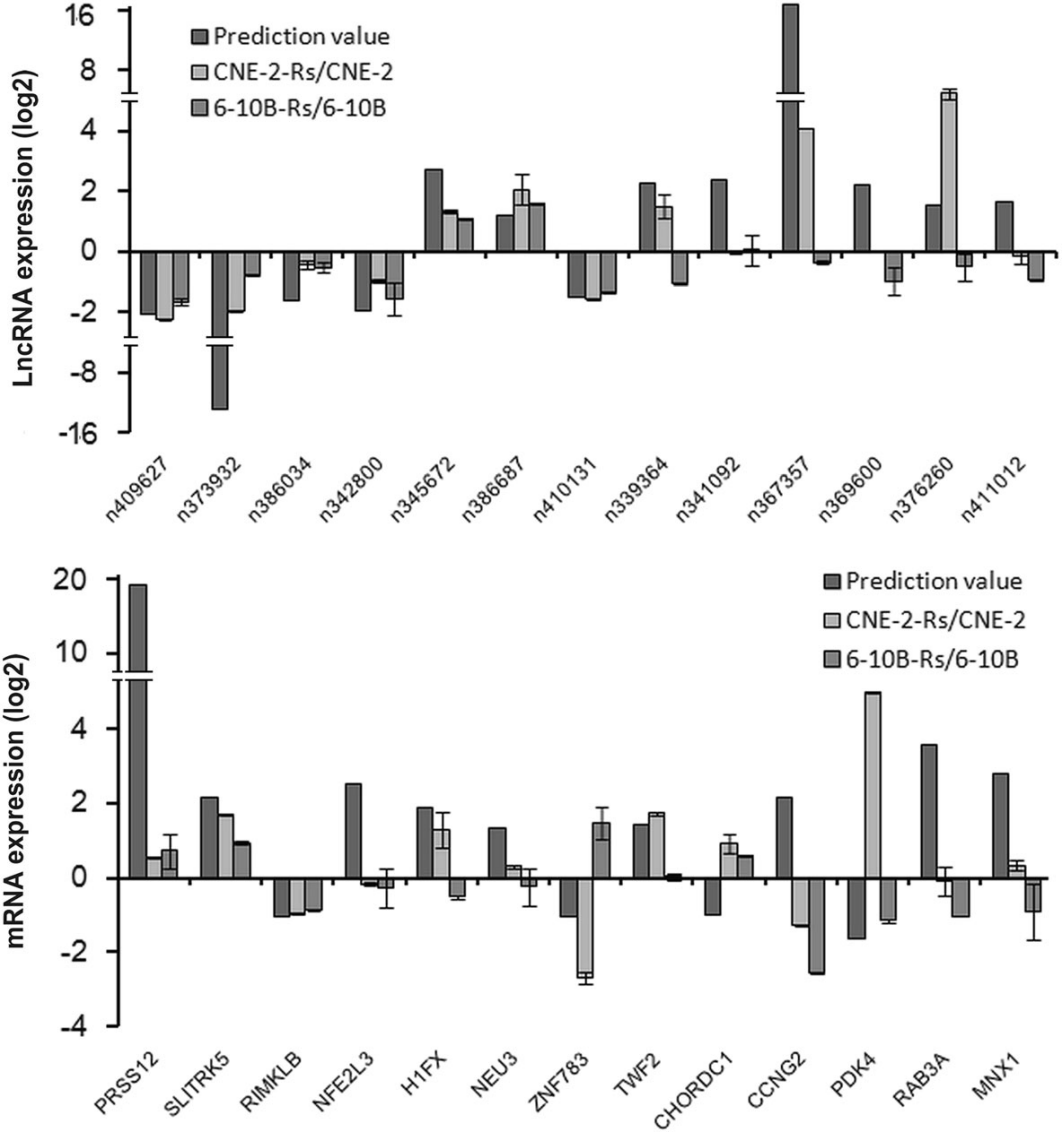
Validation of the lncRNAs and associated genes by qRT-PCR. Three pairs of lncRNAs and their associated genes exhibited corresponding changes in the prediction and both radioresistant cell lines, including lncRNA n373932 and *SLITRK5*, n409627 and *PRSS12*, and n386034 and *RIMKLB*

Based on the above prediction results, we evaluated these 13 pairs of lncRNAs and their associated mRNAs by qRT-PCR. To increase the credibility of our validation assays, we also included previously constructed radioresistant NPC 6-10B-Rs cells [11]. Our validation assays demonstrated that three pairs of lncRNAs and their associated genes exhibited changes that corresponded to the prediction in both radioresistant cell lines, including lncRNA n373932 and *SLITRK5*, n409627 and *PRSS12*, and n386034 and *RIMKLB*. Among the remaining 10 pairs, four lncRNAs (n342800, n345672, n386687, and n410131) exhibited altered expression in both radioresistant cells that corresponded to the sequencing results, but their associated genes did not correspond to the sequencing; the expression levels of the last six pairs were inconsistent with the predictions in both the lncRNAs and genes (Fig. 4a, b).

**Fig. 5:**
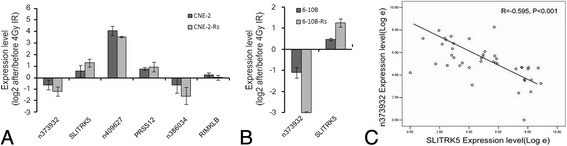
Detection of the lncRNAs and associated genes by qRT-PCR in irradiated cells and NPC cells. **a**, **b** lncRNA n373932 and *SLITRK5* had dramatic mRNA expression change in post-irradiation cell, expecially in radioresistant cells. **c** After making napierian logarithmic transformation and Bivariate Correlation analyses, we found that n373932 and SLITRK5 had a negative exprssion correlation (*R* = −0.595, *p* < 0.001, Fig. 5c)

For better understanding the molecular reaction of NPC cells to irradiation, CNE-2-Rs, 6-10B-Rs, and their parental cells were exposed to 4Gy irradiation. We chose three pairs of lncRNAs and their associated genes (n373932 and *SLITRK5*, n409627 and *PRSS12*, and n386034 and *RIMKLB*) for study in CNE-2-Rs and CNE-2. We found that lncRNA n373932 and n386034 showed dramatic expression changes in post-irradiation cells, especially in radioresistant cells. n409627 also had a post-irradiation increase but did not decrease as its sequencing result. (Fig. 5a). Among the targeted genes, only *SLITRK5 and PRSS12* showed dramatically increase with irradiation exposure, and *RIMKLB* changed slightly. Then, we found similar trends with n373932 and *SLITRK5* in 6-10B-Rs and 6-10B cells (Fig. 5b). Thus, we detected n373932 and *SLITRK5* in 43 NPC tissues via qRT-PCR analysis. The results showed that the expressions of n373932 and *SLITRK5* were negatively correlated (*R* = −0.595, *p* < 0.001) (Fig. 5c). The interactions and functions should be investigated in future studies.

## Discussion

Intrinsic and acquired radioresistance is a major challenge in the management of patients with NPC [12, 20]. In routine clinical practice, NPC tissues that exhibit radioresistance are hard to obtain because remaining NPC tissues are scarce after a full course of radiotherapy and surgery is not the first-line therapeutic choice for patients with NPC. Hence, the established radioresistant NPC cell model is the best option to study the potential role of lncRNAs in NPC radioresistance. In this study, we generated global lncRNA expression profiles associated with NPC radioresistance using next-generation deep sequencing (NGS). Using this approach, we obtained known lncRNAs and novel lncRNA candidates for further investigations into the function and mechanism of NPC radioresistance.

Conventional RNomic research has relied on microarray platforms. With the innovations in RNA-sequencing technologies and computational biology, NGS emerged and has been widely applied in RNomics research. In our lncRNA study, NGS not only allowed massive parallel analyses of genome-wide expression using microarrays but also had the advantages of calculating the absolute abundance of the transcripts, identifying variations in lncRNA sequences and discovering novel lncRNAs. Accordingly, in addition to 781 known lncRNAs, we obtained 2054 novel aberrantly expressed lncRNAs that were associated with NPC radioresistance. These lncRNAs might greatly enrich the human lncRNA pool.

Recent findings have implicated lncRNAs in the processes of cancer development and progression. Their aberrant expression confers cancer cells with the capacity for malignant transformation, growth, metastasis, and resistance to chemoagents [21]. However, few reports have investigated the role of lncRNAs in radioresistance, with the exception of the following three publications. Wang et al. found that LincRNA-p21 enhanced colorectal cancer radiosensitivity by affecting the WNT signaling pathway [22]. LncRNA BOKAS decreased the radioresistance of esophageal squamous cell carcinoma via targeting *WISP1* [23]. Reversed expression of AK94004 in NPC improved curcumin-induced irradiation damage [24]. In our current study, 781 known lncRNAs related to NPC radioresistance were obtained, few of which have been studied systematically and comprehensively.

To obtain a better understanding of the function of lncRNAs in cancer, it is important to predict and investigate the possible genes that may be regulated by lncRNAs. In most cases, researchers analyzed the expression correlations between lncRNAs and mRNAs/proteins in multiple samples (three or more samples) [25, 26]. Notably, this type of analysis emphasizes the linear correlation between lncRNAs and their associated genes and neglects the important biases caused by the complicated regulation mechanisms of lncRNAs on their potential regulatory mRNAs/proteins. Our data were obtained from two cell samples (a radioresistant cell line and its parental cell line) and were insufficient for expression correlations based on statistical analysis. Thus, we adopted two prediction strategies from the bioinformatics perspective: the antisense and the up/downstream methods. The antisense lncRNA prediction algorithm depended on the hypothesis that lncRNA transcripts were transcribed from the strand opposite that of the sense transcript of the protein-coding sequence, and consequently resulted in transcriptional and post-transcriptional suppression via a series mechanism (i.e., RNA polymerase collisions, difficulties in mRNA splice site recognition, and lncRNA-mRNA combinations) [27].

Upstream lncRNAs are defined as transcripts derived within 2 kb upstream from the transcription start sites. The 2-kb upstream region possesses a frequent distribution of human gene promoters [28]. Therefore, these lncRNAs may affect their target mRNAs at the transcriptional level by interacting with the promoter region of special mRNAs [26]. Theoretically, these lncRNAs might also inhibit or promote target mRNAs via other cis-regulated elements, such as enhancers and silencers that may located within this 2-kb upstream region, which need further validation. Our results also demonstrated that four pairs of lncRNAs and mRNAs were predicted to interact in the 2-kb downstream region by possible binding based on complementary base sequences between the lncRNAs and the mRNA transcriptional domains. However, regulation from the downstream region has not been previously reported.

Our final validation assays demonstrated that three pairs of lncRNAs and their related genes exhibited changes in radioresistant cells that corresponded to the predictions, including lncRNA n373932 and *SLITRK5*, n409627 and *PRSS12*, and n386034 and *RIMKLB*. To the best of our knowledge, no studies have linked the three validated lncRNAs (n373932, n409627, and n386034) to biological and pathological processes of human diseases. With respect to their related mRNAs, *SLITRK5* was shown to be expressed in leukemia, embryonic stem cells, subsets of endothelial cells, and neural tissues, and its dysfunction could impair corticostriatal circuitry and lead to obsessive-compulsive-like behaviors [29]. *PRSS12* (Motopsin) is a mosaic protease expressed in the central nervous system; truncation of the human motopsin gene causes nonsyndromic mental retardation [30]. *RIMKLB* is expressed at the highest level in the testis and is involved in the synthesis of β-citrylglutamate, which may play a critical role in spermatogenesis [31]. No studies have examined these lncRNAs and mRNAs in terms of cancer malignant behaviors including radioresistance, and their interactions and functions still need to be investigated and validated. In addition to the prediction strategies used in our study, other prediction strategies have been used to analyze mRNAs associated with lncRNAs [7, 18]. In fact, lncRNA also formed with thousands bases and thus has a complex secondary and tertiary structure [32]. The complicated structure of lncRNAs allows binding to proteins, RNAs, and/or DNA partners and thus participation in multiple regulatory mechanisms. These theories can also give prompts for our next of NPC radioresistance study.

## Conclusion

Radioresistance is always a in crucial factor limiting the therapeutic efficacy and prognosis of NPC patients. Here we constructed an expression profile of lncRNAs in human NPC cells and found a distinct lncRNA expression profile in radioresistant cells, suggesting that these unique noncoding transcripts might contribute to the acquisition of radioresistance in NPC. Although additional in vivo studies and clinical trials are needed to verify the lncRNAs mentioned above, our study provides important insights into novel potential treatment strategies or prognostic indicators for patients with NPC.

## Additional files

Additional file 1: Table S1. Primer sequences of the lncRNAs and mRNAs. (XLS 26 kb) Additional file 2: Table S2. The list of lncRNAs with differential expression. (XLSX 342 kb)

## Abbreviations

CT: Cycle threshold
lncRNA: Long noncoding RNA
NPC: Nasopharyngeal carcinoma
qRT-PCR: Quantitative real-time reverse transcription-polymerase chain reaction
